# Study protocol for UNICEF and WHO estimates of global, regional, and national low birthweight prevalence for 2000 to 2020

**DOI:** 10.12688/gatesopenres.13666.1

**Published:** 2022-07-19

**Authors:** Julia Krasevec, Hannah Blencowe, Christopher Coffey, Yemisrach B. Okwaraji, Diana Estevez, Gretchen A. Stevens, Eric O. Ohuma, Joel Conkle, Giovanna Gatica-Domínguez, Ellen Bradley, Ben Kimathi Muthamia, Nita Dalmiya, Joy E. Lawn, Elaine Borghi, Chika Hayashi

**Affiliations:** 1 Division of Data, Analytics, Planning and Monitoring, United Nations Children’s Fund, New York, NY, 10017, USA; 2 Centre for Maternal, Adolescent, Reproductive & Child Health, London School of Hygiene & Tropical Medicine, London, WC1E 7HT, UK; 3 Division of Data Analytics and Delivery for Impact, World Health Organization, Geneva, 1202, Switzerland; 4 Independent Researcher, Los Angeles, CA, 90245, USA; 5 Department of Nutrition and Food Safety, World Health Organization, Geneva, 1202, Switzerland; 6 Programme Group, United Nations Children’s Fund, New York, NY, 10017, USA

**Keywords:** Low birthweight, global estimates, nutrition, newborn, Bayesian modelling

## Abstract

Background

Reducing low birthweight (LBW, weight at birth less than 2,500g) prevalence by at least 30% between 2012 and 2025 is a target endorsed by the World Health Assembly that can contribute to achieving Sustainable Development Goal 2 (Zero Hunger) by 2030. The 2019 LBW estimates indicated a global prevalence of 14.6% (20.5 million newborns) in 2015. We aim to develop updated LBW estimates at global, regional, and national levels for up to 202 countries for the period of 2000 to 2020.

Methods

Two types of sources for LBW data will be sought: national administrative data and population-based surveys. Administrative data will be searched for countries with a facility birth rate ≥80% and included when birthweight data account for ≥80% of UN estimated live births for that country and year. Surveys with birthweight data published since release of the 2019 edition of the LBW estimates will be adjusted using the standard methodology applied for the previous estimates. Risk of bias assessments will be undertaken. Covariates will be selected based on a conceptual framework of plausible associations with LBW, covariate time-series data quality, collinearity between covariates and correlations with LBW. National LBW prevalence will be estimated using a Bayesian multilevel-mixed regression model, then aggregated to derive regional and global estimates through population-weighted averages.

Conclusion

Whilst availability of LBW data has increased, especially with more facility births, gaps remain in the quantity and quality of data, particularly in low-and middle-income countries. Challenges include high percentages of missing data, lack of adherence to reporting standards, inaccurate measurement, and data heaping. Updated LBW estimates are important to highlight the global burden of LBW, track progress towards nutrition targets, and inform investments in programmes. Reliable, nationally representative data are key, alongside investments to improve the measurement and recording of an accurate birthweight for every baby.

## Introduction

Reducing low birthweight (LBW) prevalence by at least 30% between 2012 and 2025 is a target endorsed by the World Health Assembly in 2012 as part of the Comprehensive Implementation Plan on Maternal, Infant and Young Child Nutrition. It also contributes to achieving the Sustainable Development Goal (SDG) 2, which aims to end all forms of malnutrition across all age groups. Birthweight is a widely used indicator of attained fetal size, with LBW defined as a weight at birth of less than 2,500g, regardless of gestational age and sex ^1^. LBW includes both live-born preterm neonates (<37 completed weeks of gestation) and live-born growth-restricted neonates (small-for-gestational-age (SGA) <10th centile of birthweight for gestational age and sex) who may be term or preterm. LBW increases the risk of neonatal and child mortality, neuro-developmental disability, stunted linear growth in childhood, and longer-term consequences of fetal programming, such as increased risk of obesity and diabetes ^2– 5^.

LBW is associated with factors contributing to preterm birth and/or fetal growth restriction such as extremes of maternal age (especially younger than 16 years of age or older than 40 years of age), multiple births, obstetric complications, maternal chronic conditions (e.g., hypertensive disorders of pregnancy), malnutrition and infections (e.g., malaria or Group B Streptococcus) ^6– 8^. In settings with high levels of fertility treatment and intensive obstetric management, including high caesarean sections rates, iatrogenic preterm birth may be an important driver of LBW ^9^. Other contributors to LBW include exposure to environmental factors, such as indoor air pollution, and tobacco and drug use ^10, 11^.

Despite the importance of LBW as a public health indicator, ongoing data challenges remain. Potential sources of bias in birthweight data that are likely to impact LBW estimates are summarized in Table 1. A major limitation of monitoring LBW is the lack of birthweight data for many of the world’s children. Many babies, especially those born outside of health facilities, are not weighed at birth; and even when weighed, low coverage of birth registration and administrative data systems, incomplete records and poor child health card retention at the household level contribute to birthweight data gaps.

**Table 1.** Potential sources of bias in low birthweight data.

Potential sources of bias in birthweight data	Likely effect * on LBW prevalence estimates
**1. Coverage of weighing: bias in newborns weighed at birth**	
1.1 Many newborns in LMIC countries are not weighed at birth, especially if born at home. These are more likely to be socio-economically disadvantaged and at higher risk of LBW.	Decreased
1.2 Extremely preterm or sick babies, those stillborn or dying soon after birth and those born around threshold of viability are the most likely to not be weighed. These babies are at high risk of being LBW.	Decreased
**2. Coverage of data system: bias in newborns included in data source**	
2.1 Low coverage of administrative data systems in many low- and middle-income countries (e.g., lower coverage of birth registration for those who die shortly after birth, missing home births, and births in private facilities even if weighed). Births in private facilities are more likely to be socioeconomically advantaged and at lower biological risk of LBW; however, high prevalence of medical interventions (e.g., caesarean sections both indicated and elective before 37 weeks) may increase risk of LBW.	Increased or decreased
**3. Loss of birthweight data: biases in missing birthweight data for newborns included in the data** ** source and weighed at birth**	
3.1 In surveys, biases in card retention (e.g., birthweight not available for babies who died and who are more likely to have been LBW) or inability to recall birthweight accurately at the time of the survey.	Decreased
3.2 Missing administrative birthweight data on the sickest babies (frequently LBW) who are transferred immediately to (and weighed in) a newborn ward.	Decreased
**4. Measurement errors: individual measurement or recording error**	
4.1 Heaping of recording of birthweight on 2500g. As definition excludes babies with birthweight exactly 2500g, those LBW newborns with birthweight near the threshold frequently heaped at 2500g.	Decreased
4.2 Errors in birthweight measurement (e.g., poorly calibrated scales, inappropriate devices), suboptimal weighing practices (e.g., clothed, or delayed weighing until >1 day after birth).	Increased or decreased
4.3 Extremely preterm or sick babies and those born around threshold of viability who die soon after birth are more likely to be misclassified as stillbirth. These babies are at high risk of being LBW.	Decreased
**5. Measurement unit error**	
5.1 Confusion in surveys where birthweights may be provided in both pounds and grams (e.g., LBW baby weighing 4.0 lbs. recorded as 4.0 kg).	Decreased
**6. Denominator calculation errors in LBW prevalence calculation**	
6.1 LBW prevalence calculated as: number with birthweight <2500 per all livebirths (whether weighed or not).	Decreased

* Decreased - the potential bias is likely to lead to a decreased LBW prevalence; Increased - the potential bias is likely to lead to an increased LBW prevalence. Source: Updated from Blencowe *et al.* ^17^ Copyright © 2019 UNICEF and World Health Organization. Published by Elsevier Ltd. This is an Open Access article under the CC BY 4.0 license.

Globally, nearly one third of newborns do not have their birthweights included in available nationally representative data sources, with major variation across regions. For example, 68.1% of newborns in Western Africa are missing birthweight data compared with just 1.4% in Europe ^12^. Furthermore, there are large disparities within countries: children born to poorer, less educated, rural, or marginalized mothers are at greater risk of missing birthweight information compared with their wealthier, more educated and urban counterparts ^13, 14^. Since these children are more likely to have LBW, estimates that do not account for missing birthweights tend to underestimate the LBW prevalence ^15, 16^. Moreover, heaping of birthweights on multiples of 100g and 500g can lead to underestimation of LBW prevalence ^13, 14, 17, 18^. Given that a child recorded as having a birthweight of 2500g is not considered LBW, rounding up of birthweights to 2500g leads to underestimation of the LBW prevalence, and consequently, to an underestimation of the care needed for these newborns. 

Despite the challenges associated with monitoring LBW, birthweight data are more likely to be collected and published in a range of populations worldwide than data on the related component indicators of preterm birth and size for gestational age. Thus, estimates based on the 2,500g cut-off allow for comparative health statistics across populations and have been the focus of several global goals since 1990 ^19, 20^. LBW reduction also has potential to contribute to other SDG targets, such as reducing neonatal and under-five mortality and preventing stunting.

In the most recent estimates (2015), a global average of 14.6% of livebirths were estimated as LBW ^17^. These estimates represented the largest systematic compilation of LBW prevalence data to date and included 1,447 country-years from 148 countries. Innovative data processing steps were introduced for these estimates, including application of data coverage and quality criteria and a revised adjustment method for survey data ^17^. To help fill data gaps, statistical regression models, including covariates of neonatal mortality rate, underweight prevalence among children aged less than 5 years, data type and region, and a country-specific random effect were used to estimate LBW prevalence. 

This protocol describes the proposed methodology and process for developing updated global LBW estimates for the period 2000–2020, which will be undertaken by the United Nations Children’s Fund (UNICEF) and the World Health Organization (WHO) in collaboration with the London School of Hygiene & Tropical Medicine (LSHTM). This protocol is informed by the Guidelines for Accurate and Transparent Health Estimates Reporting (GATHER) ^21^. We build on data sources and adjustment methods applied for the previous estimates ^17^, with data quality review enhancements, and propose a new modelling approach. We also note that this set of LBW estimates is being developed in coordination with, and will benefit from its association with, updated preterm birth estimates, for which a protocol has already been published ^22^.

## Protocol

### Project organization

A Steering Group comprised of UNICEF, WHO and LSHTM will implement this protocol. The work will be supported by an Estimates Consultative Group, comprised of global experts in LBW and preterm birth measurement, including obstetricians, neonatologists, statisticians, preterm birth researchers, modellers, and programme experts working in the measurement field. The Estimates Consultative Group will provide technical guidance on the estimation methods, and review data inputs and preliminary estimates prior to finalization. An official country consultation will be conducted with UNICEF and WHO Member States to inform them of the methodology, review preliminary national estimates and identify any additional data.

### Research ethics approval

This work is based on secondary analyses of household survey data and aggregate data from administrative sources only. The study was approved on 17th May 2021 by the London School of Hygiene and Tropical Medicine ethics review board (reference: 22858).

### Data sources

Two types of input data sources will be considered: (i) national administrative data sources; and (ii) nationally representative household surveys. National administrative data are defined as data from national systems, including civil registration and vital statistics (CRVS) systems, national health management information systems (HMIS), and birth registries. Nationally representative household surveys include Demographic and Health Surveys (DHS), Multiple Indicator Cluster Surveys (MICS), and other nationally representative surveys for which anonymized individual-level data and required variables are available. 

**Figure 1.**
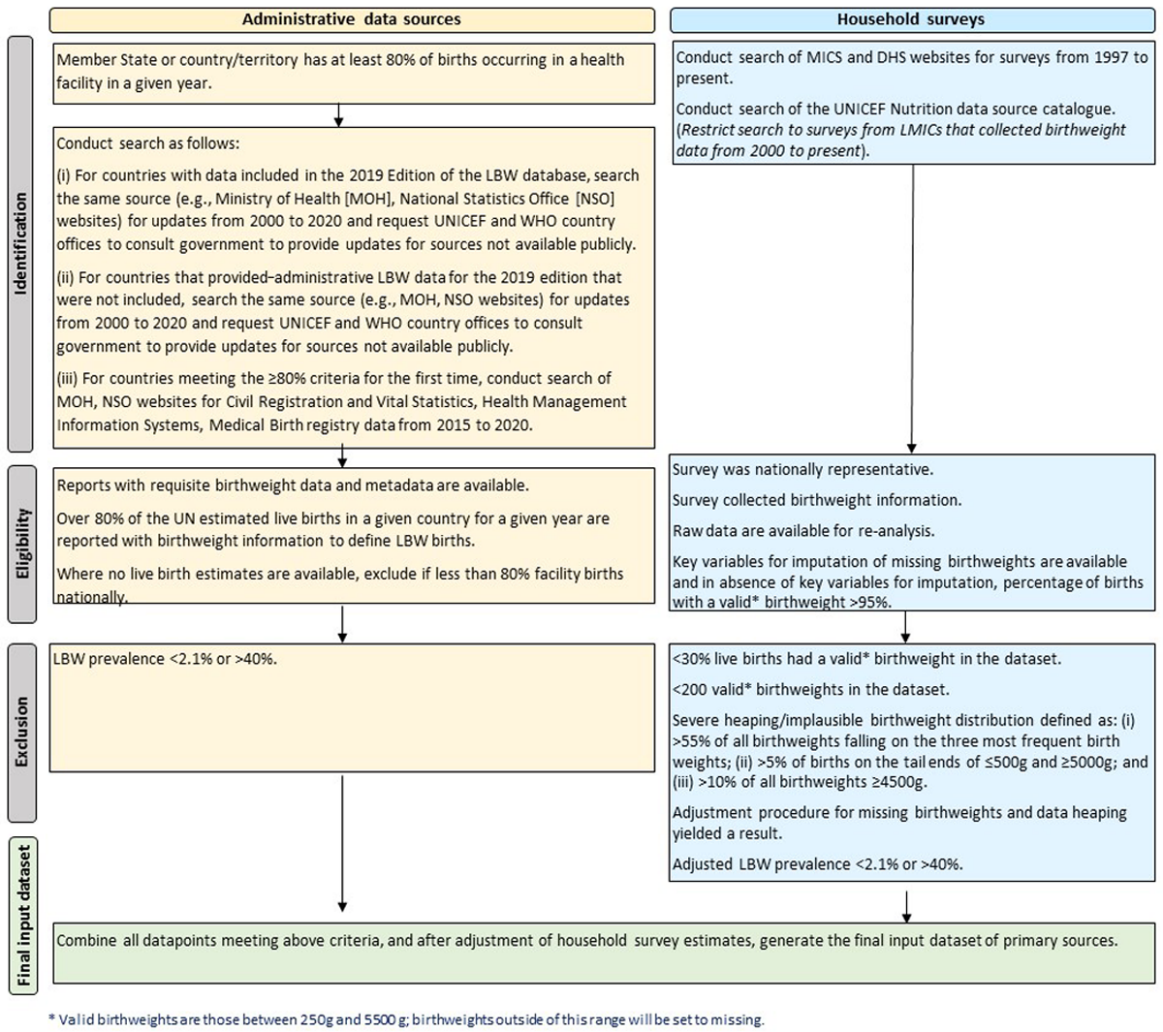
Flow diagram of the data search and review process.

Figure 1 provides an overview of the methodological steps undertaken for data search and abstraction.

### Source search strategy

***Administrative sources*.** A systematic search of Ministry of Health and/or National Statistical Office publications and datasets available in the public domain will be conducted only for countries that have at least 80% of births occurring in a health facility in a given year according to UNICEF or WHO databases ^23^. For countries that meet the search threshold, data sources and websites used for the 2019 edition of the LBW estimates will first be searched to identify more recent data from CRVS, HMIS, or medical birth registries. For countries meeting the search threshold, but without LBW data included in the 2019 edition of the LBW estimates, a web-based search will be undertaken of National Statistical Office, Ministry of Health, and other national perinatal databases to identify further data for any years from 2000 to 2020. The search terms used for English-language websites will include *birth*, *birthweight,* and *low birthweight*, with appropriate translations for non-English websites. Non-English websites will be searched by researchers who speak the relevant language. In addition to the above-mentioned methods to systematically search for LBW country data from administrative sources, UNICEF and WHO country offices will be requested to consult government counterparts for any LBW data not available in the public domain.

***Household surveys*.** We will update the database from the one used for the 2019 edition of the LBW estimates by conducting an updated search of household survey data sources to identify any missing sources. Two broad approaches will be undertaken to search for and compile updated country data on birthweight from household surveys: (i) searching the websites of DHS and MICS for surveys from 1998 to present; and (ii) searching the UNICEF Nutrition Data Source Catalogue for additional surveys that contain birthweight information from 2000 onwards from low- and middle-income countries (LMICs). 

### Data screening, review, data extraction and adjustments

***Administrative sources*.** Data will be extracted into an Excel-based data extraction form ( **Extended Data Table 1**) employing methods outlined in a guide developed for this purpose. The following variables will be extracted: country, data source, data source type, year, number of live births, number of LBW live births with sub-envelopes categorized by 500g intervals, number of live births weighing ≥2500g and number of live births missing birthweight data. LBW prevalence will be calculated as the (number of LBW live births) / (live births with a birth weight (i.e., sum of live births <2500g and ≥2500g or when ≥2500g is not available a back calculated value using reported percent LBW and number of live births weighing <2500g)) ×100. Where data on numbers of live births with a birthweight are not available, total live births will be used as the denominator, and if not available, total births (live births and still births) will be used.

The administrative data will be double extracted. The first extraction will be conducted by a single abstractor. A second abstractor will also extract all data points and any disagreement between the first and second abstractor will be resolved by a third person. For non-English data sources, review and extraction will be supported by staff that speak the relevant language. Where necessary, the relevant government agency will be contacted to help direct the reviewer to the appropriate data tables and to clarify any questions regarding the data.

***Household surveys*.** For household surveys, anonymized individual-level data will be re-analysed using STATA version 17, to produce data quality indicators (see **Extended Data Table 2),** as well as LBW prevalence estimates adjusted for missing birthweights and data heaping, with output variables described in **Extended Data Table 3**. As in the previous LBW estimates ^17^, adjustments to overcome some of the potential biases noted in Table 1 will be made, namely multiple imputation to account for missing birthweights, and fitting a finite mixture model of two normal distributions to adjust for data heaping ^17^. Birthweights reported to be <250g or >5,500g will be considered implausible based on results from the INTERGROWTH-21 ^st^ study ^24^, and will therefore be set to “missing”. For survey datasets containing the mother’s perception of size at birth, missing birthweights will be imputed using the following variables: (i) mother’s perception of size at birth; (ii) sex of child; (iii) multiple/singleton status; (iv) maternal parity; (v) maternal height; and (vi) maternal body mass index, when available. Where a mother’s perception of size at birth is not available, only the adjustment for data heaping will be performed. Following evidence from previous research ^25, 26^, five imputations will be performed for each survey, and a mixture model of two normal distributions will then be fitted to each of the five datasets of recorded and imputed birthweights. The approach provides an estimate of the proportion of birthweights <2,500g that accounts for missing values and heaping, and produces 95% confidence intervals that account for uncertainty arising from both the estimation of the parameters of the two normal distributions and from the imputation step ^27^.

### Exclusion criteria

***General exclusions for implausibility*.** All data sources with an estimated LBW prevalence of <2.1% or >40% in a given year will be considered implausible and excluded. This lower cut-off is consistent with the lowest population-based LBW prevalence among healthy women at low risk of pregnancy complications (e.g., preterm birth and fetal growth restriction) in any country from the INTERGROWTH 21 ^st^ project ^24^. The basis for the upper cut-off, consistent with that used for the 2019 edition of the LBW estimates, is from the highest population-based LBW prevalence, which was 37% ^28^.

***Administrative sources, specific exclusions*.** National administrative birthweight data will not be included for country-years where the number of live births with a birthweight is <80% of UN estimated live births ^29^, as these are unlikely to be representative of the national population.

***Household surveys, specific exclusions*.** Outputs produced during data processing and initial analysis (outlined in **Extended Data Table 2)** will be used to assess each survey against the exclusion criteria. Unweighted samples, rather than weighted samples used in the 2019 Edition of the LBW estimates, will be used to align with methods applied for data quality review of other nutrition indicators based on recent global guidance ^30^. Consistent with the previous LBW estimates ^17^, surveys will be excluded if any of the criteria listed below apply.

- Unavailability of the mother’s perception of size at birth variable, except in cases where >95% ^1^ of live births have a valid ^2^ birthweight.
- <30% ^3^ of live births have a valid ^2^ birthweight in the dataset.
- <200 ^4^ valid ^2^ birthweights are available in the dataset.
- There is severe heaping / implausible birthweight distribution, which we define as:
  i. >55% of all birthweights falling on the three most frequent birthweights (e.g., if 3,000g, 3,500g and 2,500g were the three most frequent birthweights, these three birthweights could not make up more than 55% of all birthweights in the dataset)
  ii. >5% of birthweights on the tail ends of ≤500g and ≥5,000g
  iii. >10% of birthweights ≥4,500g

### Data quality assessment

Administrative data which pass the 80% threshold will be assessed using quality indicators across four dimensions adapted from the WHO data quality review framework ^31^. The four dimensions are (i) availability of time series data; (ii) availability of aggregate data to assess data quality; (iii) internal consistency and plausibility; and (iv) external comparability and plausibility. This data quality review will inform subsequent statistical analyses and sensitivity analyses, and will help quantify and adjust for potential biases and limitations of the LBW estimates.

Table 2 summarizes the overall approaches that will be taken to minimize the risk of bias outlined above.

**Table 2.** Risk of bias assessment and potential approaches.

	Criteria	Potential biases	Proposed approach admin data	Proposed approach survey data
1.	Population representativeness of available birthweight data	Biases in newborns included in data source and biases in birthweight availability for included newborns. (Table 1 – Potential biases 1,2,3)	Exclude if the total births with a weight in the data source is <80% of UN-estimated population of live births. Consider sensitivity analyses.	Include only surveys designed to be nationally representative. Only include surveys with valid birthweights for ≥30% of births, and for those, undertake multiple imputation to impute birthweight data for included newborns with missing birthweight. Set a stricter inclusion criterion of ≥95% requiring a valid birthweight for surveys where multiple imputation is not possible.
2.	Birthweight distribution	Biases due to missing birthweight for very sick babies and those born around the threshold of viability (Table 1 – Potential biases 1,3)	Categorize data where possible into LBW subgroups % for very low birthweight, extremely low birthweight and <500g. Review distributions and identify data with evidence of under-capture of those <1,000g. Consider adjusting these data or sensitivity analysis based on excluding these data.	Multiple imputation to impute birthweight data for included newborns with missing birthweight. Very sick or small babies who die immediately after birth may not be captured in the birth history at all. Thus, consider sensitivity analysis based on excluding data points with evidence of under-capture of those <1,000g.
3.	Measurement errors due to heaping	Heaping of recorded birthweight on 2,500g. (Table 1 – Potential biases 4)	Consider use of administrative data birthweight heaping index for countries with available information to identify indicators of countries that have higher and lower prevalence of heaping. Use model terms for categories of administrative data in the Bayesian model to adjust data in countries that are expected to have high heaping.	Exclusion of surveys with extreme heaping (>55% of all birthweights falling on the three most frequent birthweights and <5% of births on the tail ends of ≤500g and ≥5,000g) Also, heaping adjustment undertaken as part of the pre- modelling data processing.
4.	Measurement errors due to misclassification of live births as stillbirths	Most likely in babies around the perceived thresholds of viability, which vary by context (Table 1 – Potential biases 4)	Methods detailed above on birthweight distribution to attempt to identify missing babies around the threshold of vulnerability.	Misclassified newborns will be missing from the survey dataset. Methods detailed above on birthweight distribution to attempt to identify missing babies around the threshold of vulnerability.
5.	Measurement unit error	Confusion in surveys where birthweights may be provided in both pounds and grams (Table 1 – Potential biases 5)	Not applicable	Exclusions based on >10% of all birthweights ≥4,500g. ^5^
6.	Incorrect denominator used	For example, where a large number of newborns in the data source did not have a recorded birthweight and the denominator used includes all newborns in data source, rather than all newborns with a birthweight in the data source. (Table 1 – Potential biases 6)	Re-calculate LBW prevalence estimates using the correct denominator, if available; explore other approaches to account for bias if not.	Re-calculate all LBW prevalence estimates.

Note: Any remaining error will be captured by model terms for non-sampling variability

### Statistical analysis and modelling

After eligibility and exclusion criteria are applied to the extracted and re-analysed data, one dataset of survey and administrative estimates will be compiled. In compliance with GATHER guidance, the following details of all included data sources will be made publicly available: reference information or contact name/institution, population represented, data collection method, year(s) of data collection, and sample size, as relevant.

*Step 1: Covariates selection for modelling*

**Figure 2.**
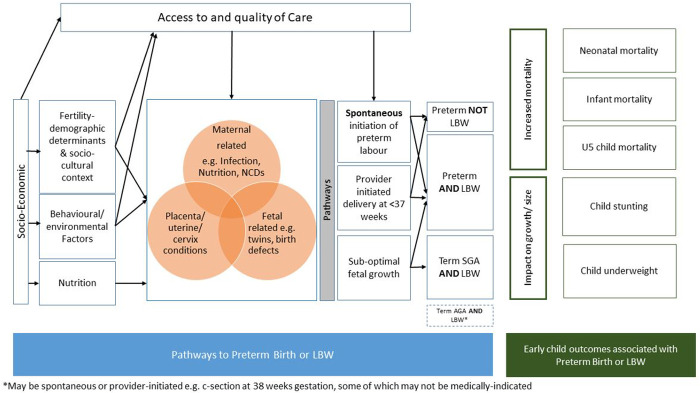
Conceptual framework for the identification of potential covariates for use in the low birthweight estimates. This framework has been informed by previous publications ^32– 34^.

The development of the models for the LBW estimates will utilize country-level covariates available from the United Nations and other sources. Covariates for inclusion will be selected a priori using a three-step approach as follows: (i) identifying plausible predictors and outcomes for LBW based on a conceptual framework ( Figure 2); (ii) assessing data availability and data quality of potential covariates time series; and (iii) assessing correlation between covariates, correlation of covariates with LBW, and clustering analysis to select one covariate within each cluster based on correlation levels and data availability.

Plausible predictors of LBW were identified through construction of a conceptual framework based on biological plausibility and risk factors ( Figure 2) using existing frameworks in the literature ^32– 35^. The conceptual framework illustrates the pathways to LBW and the relationship between socioeconomic and demographic factors, maternal nutrition and health status, and access to health care. It also shows the links between early childhood outcomes that are associated with LBW, which will be considered as potential predictors of the model, including child malnutrition (e.g., stunting and underweight among children under 5 years of age) and early child mortality (e.g., neonatal mortality rate, infant mortality rate).

Potential covariates across six domains: (1) socio-economic, demographic, fertility, and cultural factors; (2) nutritional, behavioural, and environmental factors; (3) maternal conditions (including infections); (4) fetal or placental conditions; (5) health care-related factors (markers to access to care); and (6) early childhood outcomes associated with LBW/preterm birth are presented in Table 3.

**Table 3.** Covariates for potential inclusion in the modelling analyses.

Domain	Potential covariate
**Socio-economic, demographic,** ** fertility, and cultural factors**	Gross National Income
	Gross Domestic Product
	GINI coefficient
	Adult Female Literacy Rate
	Mean years female education
	Adolescent Birth Rate
	Total Fertility Rate
	General Fertility Rate
	Modern contraceptive rate prevalence
	Proportion of live births to mothers aged 35 and older
	Urban Population
**Nutritional, behavioural, and ** **environmental factors**	Adult Female Smoking Rate
	Indoor air Pollution
	Outdoor air pollution
	Adult Female Body Mass Index (Mean)
	Underweight women of reproductive age
	Overweight women of reproductive age
	Maternal Anaemia
	Adult Female Substance Use
	Intimate Partner Violence
**Maternal conditions (including** ** infections)**	Maternal Mortality Rate
	Adult Female HIV Prevalence
	Malaria Incidence ( *P. falciparum* Parasite Rate)
	Insecticide-treated nets coverage
	Adult female Syphilis prevalence
	Gestational Hypertension
	Gestational Diabetes
	Maternal Depression
**Fetal or placental conditions**	Twinning
	Birth Defects
	Growth restriction
**Health care-related factors** **(Markers of Access to care)**	Antenatal Care Attendance (Four or more times)
	Skilled Birth Attendance
	Facility Birth Rate
	Caesarean Section Rate
**Early childhood outcomes ** **associated with LBW**	Neonatal Mortality Rate
	Stunting prevalence in children under 5 years
	Underweight prevalence in children under 5 years

Data availability will be assessed through consultation with WHO and UNICEF colleagues and a targeted search of webpages of United Nations organizations (e.g., WHO Global Health Observatory, UNICEF, United Nations Population Division) and academic groups (e.g., International Health Metrics and Evaluation (IHME)).

For potential covariates with no existing time series estimates available, UNICEF and WHO databases will be searched for empirical data available for these variables. Where comparable, but incomplete, time series data are located for a given covariate, a new time series will be generated using a standard approach for in-filling and extrapolation consistent with previously used approaches ^36^. Namely, for countries with some empirical data, linear interpolation and constant backwards and forwards extrapolation will be used. For countries with no empirical data, values will be imputed using a regression based on geographic region and country’s lag distributed GDP and World Bank country income classification. Finally, for all countries, smoothed time series will be generated using a 7-year average for model prediction.

Given that ideal covariates for LBW would be comprised of estimates from primary data sources (i.e., not modelled using covariates) for all years from 2000 to 2020, potential covariates will be assessed considering data source, number of empirical data points available by country and methods used to produce time-series including any modelling, in-filling, smoothing, extrapolations, or any other data manipulations.

Finally, exploratory analysis will be undertaken to observe correlations between potential covariates and for each covariate with LBW. To select a parsimonious set of covariates that avoids model overfitting, cluster analyses of all covariates will be undertaken with the aim of having distinct clusters from which only one covariate per cluster will be selected for inclusion in the modelling. The selection of covariates within a cluster will be based on covariates that have the highest correlation with LBW or covariates with data for most country-years in cases where the correlation coefficients are deemed not to be that different from the covariate with the highest correlation though incomplete data.

*Step 2: Development of a model to estimate low birthweight prevalence*

A Bayesian multilevel-mixed regression model will be developed to estimate LBW prevalence at national level. Analysis will be conducted using RStudio 2021.09.0+351 "Ghost Orchid" Release, and the RJAGS RJAGS, R2JAGS, RSTAN packages. The model will process all country-years with ‘available data’, including the regional ^37^ intercepts and country-specific intercepts and slopes, generating LBW estimates for all country-years. The model will include terms for data source characteristics ( *e.g.,* survey versus administrative data, and/or measures of data quality). Temporal variability will also be considered at the country and regional level. Covariates selected from *Step 1* will be included in the model. LBW will be modelled on the logit scale to ensure that LBW prevalence estimates and confidence intervals obtained from the fitted model are within a plausible range (i.e., between 2.1% and 40%). In case of an implausible and unexpected direction of one or more of the included covariates based on the estimate of regression coefficients, the covariate within a cluster that is next in rank based on the correlation coefficient with LBW will be selected, and so on.

*Step 3: Generating estimates of LBW prevalence and trends*

Annual estimates of national LBW prevalence from 2000 to 2020 will be predicted from the Bayesian multilevel-mixed regression model developed in step 2, for all countries, including for country-years with data and country-years without useable data, or no data at all.

Various sensitivity analyses will be performed comparing: the final LBW model (with and without covariates to evaluate the contribution of the covariates used), and a model that includes additional covariates used in the 2019 edition of the LBW estimates.

*Step 4: Presentation of results*

Country-level point estimates with the 10 ^th^ and 90 ^th^ percentiles for uncertainty intervals around the estimate will be presented. A specific review of data availability and estimates for the year 2020 will be applied to assess any effects of the COVID-19 pandemic and response; estimates for 2020 will be published depending on the outcome of the assessment. Only national estimates for those countries contributing at least one eligible data point in the estimation period will be published. Nevertheless, estimates derived for all countries and years will contribute to the regional and global estimates.

### Access to data

In compliance with GATHER guidance ^21^, the final LBW estimates with uncertainty intervals will be published online through the WHO Global Health Observatory and UNICEF Data website alongside the complete database of input data used to develop modelled estimates and relevant code. The following information will be made publicly available for all included data sources: reference information or contact name/institution, population represented, data collection method, year(s) of data collection and sample size, as relevant.

### Dissemination

This work will result in publication of global, regional, and national LBW prevalence estimates for the period of 2000–2020 in an open-access peer-reviewed journal. We will also publish the final protocol, database and LBW prevalence estimates online through the WHO Global Health Observatory and UNICEF Data website, according to GATHER ^21^, as described in the previous section.

## Discussion and conclusion

The development of LBW prevalence estimates is critical for all countries and yet there are challenges anticipated in this work that we have noted as part of this study protocol.

Firstly, with regards to population representativeness, national data sources are often incomplete or unavailable, particularly in LMICs. National administrative data sources may miss marginalized or vulnerable groups (e.g., those in humanitarian settings, indigenous populations) who may face greater risks of LBW. We will not include administrative data from data systems with low population representativeness (covering <80% of national livebirths); while some of these countries will have nationally representative survey data included, others may have no LBW input data. 

Biases may arise due to missing birthweight data on newborns around the threshold of viability – the smallest and most preterm newborns. Methods for analysing individual-level survey data will partially address missing birthweights. For administrative data, we will consider adjusting data points with evidence of missing birthweights and/or under-capture of those <1,000g. However, this assessment will be limited to countries that capture and report such data, noting that those most prone to biases are also most likely to lack such information. 

Potential sources of bias, and approaches to address these, have been considered above ( Table 2), and sources with high levels of missing data will be excluded. It is not possible to adjust for all biases due to measurement errors. Whilst adjustments for heaping will be applied to survey data, there are currently no established methods for adjusting aggregate data from administrative sources. Evidence on the extent of heaping in administrative data are limited to a subset of countries, meaning that systematic adjustment is not possible nor is it possible to adjust the administrative estimates for heaping at an individual level since microdata were not obtained. Instead, other data quality indicators that are available for the administrative data will be used as a proxy to inform the structure of the Bayesian model to account for these factors. Challenges arising from the low quality of some data are compounded by absence of clear, internationally harmonized guidelines on how to assess the quality of birthweight data. In the future, methods to adjust for incomplete or low-quality administrative data may help overcome these biases.

The work described in this protocol will be used to generate estimates of LBW prevalence at global, regional, and national levels for the period of 2000 to 2020. This protocol builds closely on the methodology used for the 2019 edition of the LBW estimates ^17^. In successive estimation rounds, increases in data capture for administrative systems will allow for an expanded number of national data points from more countries to be included in the estimation work. The availability of birthweight data with sufficient coverage from household surveys from LMICs is also expected to improve over time (UNICEF and WHO, 2019); however, some data gaps remain in recent years due to delays related to the COVID-19 pandemic. The increase in the number of countries with data, as well as the quantity of data points per country, are expected to improve the estimates overall, although some data quality issues, such as heaping of birthweights, may not improve at the same pace.

The current round of LBW prevalence estimates for the period of 2000 to 2020 will be critical for targeting programs that aim to reduce LBW prevalence over time. These estimates will also guide the refinement and implementation of nutrition and health policies, inform resource allocation within health systems, and help assess the impact of nutrition and newborn survival interventions and their respective redesign.

## Study status

The research is currently underway with the administrative and survey data searches, extraction and re-analysis completed in June 2022, the model developed and tested from January to July 2022, country consultation set to begin in August 2022 and final estimates planned for release in the fall/winter of 2022.

## Data availability statement

### Underlying data

No underlying data are associated with this protocol.

### Extended data

figshare. Extended data table1 admin data abstraction template.xlsx. https://doi.org/10.6084/m9.figshare.20113040.v1 ^38^

This project contains the following files:

- Extended data table1 admin data abstraction template.xlsx (data extraction form for low birthweight and preterm birth estimates)

figshare: Extended data tables 2 and 3 LBW protocol.docx. https://doi.org/10.6084/m9.figshare.20113043.v2 ^39^

- Extended data tables 2 and 3 LBW protocol.docx (variable description for household survey reanalysis outputs for data quality review)

Data are available under the terms of the Creative Commons Attribution 4.0 International license (CC-BY 4.0).

## Reporting guidelines

No standard reporting guideline exists for protocols for global estimates. Final estimates will be reported in accordance with the GATHER statement ^21^.

## Footnotes

^1^ Data sources with ≥95% of livebirths with a valid birthweight, but no data on mother’s perception of size of birth, will be adjusted for heaping only, and their inclusion will be assessed in a sensitivity analysis.

^2^ Valid birthweights are defined as those falling between 250g and 5500g; birthweights falling outside this range are set to ‘missing’.

^3^ Note that coverage of livebirths weighed is among births with a valid birthweight for surveys and is much lower (≥30%) than required for administrative data sources (≥80%) because raw data are available for surveys, allowing multiple imputation of missing birthweights by use of other covariates from the survey.

^4^ The criteria requiring at least 200 birthweights and 30% of births with a birthweight in the dataset are intended to allow a sufficient sample for application of the adjustments for missing birthweights and data heaping.

^5^ The most common source of confusion is between lbs and kg, such as a baby just below the LBW threshold weighing 5.5 lbs but recorded as 5.5 kg). This criterion thereby excludes surveys with a large amount of unit confusion.
