# Supplementary material for: Study protocol for UNICEF and WHO estimates of global, regional, and national low birthweight prevalence for 2000 to 2020
Source: Gates Open Res. 2022 Jul 19;6:80. [Version 1] doi: 10.12688/gatesopenres.13666.1 (PMC10229761; doi:10.12688/gatesopenres.13666.1)
Supplement: Peer review history

# Peer review: Reviewer response for version 1

**DOI:** 10.21956/gatesopenres.14951.r33248

**Published:** 2023-06-30

**Authors:** Aloka L Patel, Esther Lee

**Affiliations:** 1 Department of Pediatrics, Rush University, Chicago, IL, USA

The authors outline a meticulous and comprehensive study protocol to update low birth rate prevalence for 202 countries between 2000 to 2020. The overarching goal of this protocol is to estimate the current global LBW prevalence in order to create programs and track progress towards targets to ultimately achieving SDG 2 goals. We want to recognize the exemplary work by the authors in putting together this protocol. We specifically found the handling the potential sources of bias in birthweight data to be comprehensive and thoughtful. We know that data driven programs and effort lead to more targeted and meaningful improvement, and this protocol will provide the foundation for future world impact on mitigating morbidity and mortality due to LBW.

 We have some thoughts and clarifying questions that may help guide the authors to improve the organization and streamline the protocol to be more easily followed by those with less technical statistical knowledge.

 Page 3: paragraph 1: after “live-born growth-restricted neonates add "i.e". before (small-for-gestational-age (SGA))

- The term growth restriction is often used with IUGR (Intrauterine growth restriction), so it would provide clarity to add the "i.e" to show that SGA is an example of “live-born GR'

Page 6 “For survey datasets containing the mother’s perception of size at birth, missing birthweights will be imputed using the following variables: (i) mother’s perception of size at birth; (ii) sex of child; (iii) multiple/singleton status; (iv) maternal parity; (v) maternal height; and (vi) maternal body mass index, when available. Where a mother’s perception of size at birth is not available, only the adjustment for data heaping will be performed. Following evidence from previous research25,26,”

- Understanding that gestational age (GA)/Ultrasound guided accurate GA may not be widely available in LMICs, we would recommend factoring in infant’s GA or estimated GA when available?

Page 7: For countries with no empirical data, what is the rationale for inclusion with inter/extrapolation of data versus omitting that country? We understand that the generated data could serve to be a valuable reference for future data collection. However, it would be valuable to understand the reasoning.

- Also, we suggest considering sensitivity analysis with including/excluding countries without empirical data.

Page 8: criteria 2: consider sensitivity analysis based on excluding data points with evidence of under-capture of those <1,000g---Yes, we would support this

 Page 9, Fig. 2: Consider writing out "U5"= under 5 child mortality. The font could be mistaken for US (United States).

 Page 10: Table 3 “Early childhood outcomes associated with LBW”

- The rationale behind including early childhood outcomes associated with LBW is unclear. Is it due to impact of the potential covariates on maternal health due to early life experiences and subsequent impact on fetus and neonate? Or are these covariates serving as markers of higher rates of LBW outcomes based on data from the geographical area?

Is the study design appropriate for the research question?

Yes

Is the rationale for, and objectives of, the study clearly described?

Yes

Are sufficient details of the methods provided to allow replication by others?

Yes

Are the datasets clearly presented in a useable and accessible format?

Not applicable

Reviewer Expertise:

Dr. Patel: neonatology, nutrition, disparities, clinical and economic outcomes, growth.  Dr. Lee: neonatology, global health.

We confirm that we have read this submission and believe that we have an appropriate level of expertise to confirm that it is of an acceptable scientific standard, however we have significant reservations, as outlined above.

# Peer review: Reviewer response for version 1

**DOI:** 10.21956/gatesopenres.14951.r33402

**Published:** 2023-06-09

**Authors:** Zachary Vesoulis

**Affiliations:** 1 Washington University in St Louis, St. Louis, Missouri, USA

In this study protocol, Krasevec *et al.* describe a multipronged approach for broad estimation of low birthweight (LBW) incidence across 200 countries. Accurate assessment of LBW incidence is an important foundation of reducing infant and child mortality and a key measure for world health initiatives.

 In this protocol, the authors detail a strategy for the acquisition and handling of data from a variety of administrative and institutional sources along with a statistical approach to handle anticipated challenges.

 This is an important project and a clearly written protocol and the authors should be commended for taking on this initiative. They have accurately identified several key barriers including missing data, heaping of data, and lack of reliability in the data. For some of these problems (especially heaping) they present sufficient statistical methods to overcome the challenge.

 The problem of missing data looms large and will require significant effort on the part of the team. In this particular case, missing data are truly missing, making measures as simple as a denominator (much less accurate birth weights) difficult to ascertain. The authors have provided thresholds of data missingness and an imputation strategy, however I anticipate that the real data will not be missing in such a "neat" fashion. I would suggest that they consider partnership with organizations or individuals in regions where there is a high degree of missingness to provide direct estimation of birth weights in small representative samples to be used as validation for the more general statistical strategies.

 I am also concerned about overreliance on web based sources of data and the primarily English-language focus. The authors note that collaborators who speak "the relevant language" will participate in the study, however it is unclear the breadth of language expertise available and additional bias may be introduced through language or record accessibility.

 Finally, the usage of household survey data provides an excellent alternative source of "truth" for the purposes of the study and I appreciate the systematic approach to evaluating the survey output to ensure validity. However, it is unclear to this reviewer how standardized or available the surveys are and how readily the analytic framework can be applied to broad survey design. Unless the surveys are standardized forms, this heterogeneity will likely prove challenging and result in additional missing data, particularly when compared to vital statistics which are more readily comparable.

Is the study design appropriate for the research question?

Yes

Is the rationale for, and objectives of, the study clearly described?

Yes

Are sufficient details of the methods provided to allow replication by others?

Yes

Are the datasets clearly presented in a useable and accessible format?

Not applicable

Reviewer Expertise:

Prematurity, outcomes research, brain injury, neurodevelopment

I confirm that I have read this submission and believe that I have an appropriate level of expertise to confirm that it is of an acceptable scientific standard, however I have significant reservations, as outlined above.

# Peer review: Reviewer response for version 1

**DOI:** 10.21956/gatesopenres.14951.r33246

**Published:** 2023-06-02

**Authors:** Karen J. Mathewson, Christina A. Brook

**Affiliations:** 1 Department of Psychology, McMaster University, Hamilton, Ontario, Canada

The authors outline a study protocol involving up to 202 countries that is designed to estimate the prevalence of low birth weight (LBW) between 2000 and 2020 at world-wide, regional, and national levels. The protocol focuses on two major sources of data, namely, national repositories of administrative data (provided the available birthweight data account for ≥ 80% of UN-estimated live births for that country/year), and birthweight data from population-based household surveys. Analyses include quality and bias assessments, comprehensive extraction of birth-weight related information from national repositories, and the development of regression models from household survey data. Sensitivity analyses also will be conducted to evaluate the contributions to the models from covariates in six domains (demographic/socio-economic factors, nutritional/environmental factors, maternal health conditions, fetal or placental conditions, factors related to access to care, and early childhood outcomes such as neonatal mortality and childhood stunting). Updated data on LBW rates are essential for evaluating the global burden of LBW and programs designed to reduce these rates, in accord with one of the Sustainable Development Goals set by the World Health Assembly in 2012, which is to reduce rates of LBW by at least 30% by 2025.

 This paper presents an ambitious protocol to obtain updated estimates of LBW prevalence at national, regional, and global levels over the twenty-year period ending in 2020. The authors are to be highly commended for their rigour in compiling a statistical approach for analyzing such a complex dataset, including attention to the representativeness of the data and potential sources of bias in the data. Below are some thoughts and queries that might be helpful in clarifying the protocol:

- Carrying the complex design from beginning to end of the paper was somewhat challenging for anyone inexperienced with such protocols. This could be improved with some re-organization of the structure of the text. An overall objective could be inserted after the Introduction. Perhaps the switching back and forth in the *Protocol* section between how the national administrative data and survey data will be handled could be avoided by first describing all of the plans for the national data (screening, extraction exclusions), and then all of the plans for the survey data (screening, analyses, exclusions).  
- It would be helpful if the authors would provide brief, explicit rationales for some of the decisions they made. For example, although they describe the household surveys as “nationally representative”, it becomes clear that the point of using the surveys is that they are an essential secondary data source when national administrative data are unrepresentative of their populations due to missing data. The reason for using survey data could be made explicit. Second, how was the 30% threshold arrived at for inclusion of survey data? In another example, it is not clear why fitting imputed data to a mixture model of two distributions is necessary or important when adjusting for data heaping. Finally, what validation is available for selecting the variable “mother’s perception of newborn size”? There may be other examples.
- The authors propose a mixed effects model (also known as a multilevel model) to model incomplete LBW data from 202 countries. There are many strengths to this design, including the use of Bayes estimation, given the likelihood of nonnormality in sample(s’) distributions and the complexity of the data, as well as the use of mixed effects to account for data dependency. It would be helpful to have a summary statement at the beginning of the statistical analysis section that outlines which analysis will be undertaken at each geographical level (national, regional, or global).
- Another strong point in favour of the modeling design for the survey data is the inclusion of carefully selected covariates (through EFA and a conceptual framework) that account for known associations with LBW status. One concern, however, is the intent to use early childhood outcomes as covariates. Perhaps we have misunderstood the intended design, but we wonder about the validity of using outcomes such as undernutrition in childhood to predict birthweight status, when temporal precedence would indicate that these outcomes postdate LBW. The authors indicate that such early childhood outcomes are often correlated with LBW, but more justification is needed.
- It was challenging to understand the goals of the statistical analyses of the survey data in terms of predictors and outcomes. Step 1 (covariate selection) is clear. Perhaps we misread the text, but it is not clear how a Bayesian multilevel model “will be developed to estimate LBW prevalence at a national level” at Step 2, which is then used to predict annual estimates of national LBW prevalence for all countries in Step 3.
- The sensitivity analyses will be very helpful. In addition to evaluating the contribution of the various covariates to LBW estimates, will significant covariates ultimately be used to inform policy, or for some other purpose?
- It would be useful to the reader to have a figure depicting a basic Bayesian multilevel-mixed regression model, with direction of effects, to clarify some of the specific relations that will be tested. The conceptual framework in Fig. 2 does not carry all of this relational information. It would also be helpful to have a simple paragraph describing the steps in the modeling process from gathering data to estimating model parameters of interest (i.e., gather data, assess at the national level for relations one, two and three, assess at the global level for relations one two and three etc.)
- It was not entirely clear how missing data was being handled statistically at each level of analysis. Do any of the statistical approaches determine whether *data is missing at random* (MAR) versus NMAR, which in turn dictates how missingness is handled? It is difficult to imagine that the samples will be free of data that are *not missing at random* (NMAR), which may produce biased rather than “true” estimates (e.g., Muthén & Asparouhov, 2011), in addition to the problems identified by the authors in Table 2.
- It would be reassuring to have a separate section on expected methodological limitations of the protocol.

Minor issues:

- What does “temporal variability” at the country and regional levels refer to, at the end of p. 7? 
- Please name the “standard approach for infilling and extrapolation consistent with previously-used approaches” alluded to in reference 36 (p. 7)? This sentence could be clearer.

Is the study design appropriate for the research question?

Partly

Is the rationale for, and objectives of, the study clearly described?

Yes

Are sufficient details of the methods provided to allow replication by others?

No

Are the datasets clearly presented in a useable and accessible format?

Not applicable

Reviewer Expertise:

kjm: extremely low birth weight; psychophysiology                cab: statistical analyses; statistical modelling

We confirm that we have read this submission and believe that we have an appropriate level of expertise to confirm that it is of an acceptable scientific standard, however we have significant reservations, as outlined above.

# Peer review: Reviewer response for version 1

**DOI:** 10.21956/gatesopenres.14951.r33099

**Published:** 2023-05-30

**Authors:** Hong-tian Li, Hong-zhao Yu, Yang Liu

**Affiliations:** 1 Peking University, Beijing, Beijing, China

The protocol describes in detail the data sources, data processing, and modeling approach used to estimate the global low birth weight (LBW) prevalence from 2000 to 2020. The proposed methodology offers insightful solutions for dealing with potential biases in LBW estimates. Below are some suggestions for further optimization.

 Firstly, the authors mentioned to use a novel modeling strategy in comparison to the 2019 study, but did not describe in detail the advantages of the new strategy. Readers may be interested to know the reason for that.

 Secondly, it is reasonable to exclude implausible data points prior to data synthesis, but a 40% upper limit may not be appropriate across all settings. Specifically, for developed countries, this threshold may be too high, and may result in inclusion of unlikely data points for these countries (i.e., 39%). Is it possible to set exclusion criteria tailored to specific regions based on the 2019 study?

 Thirdly, it may need to clarify whether only one data point per year per country will be included and incorporated into statistical modelling.

 Fourthly, more detailed descriptions regarding covariate selection would be beneficial. For example, the authors mentioned that "or covariates with data for most country-years in cases where the correlation coefficients are deemed not to be that different from the covariate with the highest correlation though incomplete data." It would be beneficial to set explicit criteria in advance to determine what levels of difference qualify as no difference. Additionally, on page 7, the authors stated that "Finally, for all countries, smoothed time series will be generated using a 7-year average for model prediction." However, the reasons for using the 7-year average for model prediction is unclear.

 Finally, in Figure 1 and Table 1, abbreviations such as MOH and NOS are used without explanation. The exclusion criteria for household surveys in Figure 1, "adjustment procedure for missing birthweights and data heaping yield a result", are not immediately self-explanatory.

Is the study design appropriate for the research question?

Yes

Is the rationale for, and objectives of, the study clearly described?

Yes

Are sufficient details of the methods provided to allow replication by others?

Yes

Are the datasets clearly presented in a useable and accessible format?

Yes

Reviewer Expertise:

Perinatal Epidemiology

We confirm that we have read this submission and believe that we have an appropriate level of expertise to confirm that it is of an acceptable scientific standard, however we have significant reservations, as outlined above.

# Peer review: Reviewer response for version 1

**DOI:** 10.21956/gatesopenres.14951.r33247

**Published:** 2023-05-30

**Authors:** Mitsuhiro Haga

**Affiliations:** 1 Department of Neonatology, Saitama Medical Center, Saitama, Saitama Prefecture, Japan

**General comments:**

 Thank you very much for allowing me to review such an outstanding protocol paper. The authors designed a detailed protocol for estimating the prevalence of low birthweight infants worldwide. One of the major challenges for collecting populational data is under and/or inaccurate reporting, which is much more prevalent in the most vulnerable populations. The study protocol is clearly addressing the biases involved in the public health study. The most striking part of this study is that the authors will review and extract non-English data sources with the support of staff who can speak relevant languages. Not every study in the field of public health does such an inclusive methodology. I admire the passion and thoughtfulness of the authors. Their plan for data extraction and statistical analyses is plausible and reasonable. I have a few minor comments that the authors can be answered quickly.

**Specific comments:**

1. Statistical analysis:

 The authors are going to create a Bayesian multilevel-mixed regression model predicting the prevalence of low birthweight infants. The R packages they will use in this study are specified as “RJAGS RJAGS, R2JAGS, RSTAN packages.” in the manuscript (P7, C2, L49–50). However, I cannot find such R packages. Would they be “rjags, R2jags, and rstan”? It would be much more desirable if the authors added citations of those packages as references.

2. Statistical analysis:

 It seems the authors will perform statistical analysis with JAGS and Stan via R software. I am curious to know how the authors are going to use these two packages. I understand that JAGS and Stan are used for creating a Bayesian multilevel model. Are the authors going to use JAGS and Stan for different purposes? Otherwise, are they going to use the two as cross-validation?

3. Figure 1:

 It would be much more reader-friendly if the authors specified the full spelling of MICS, DHS, and LMICs.

4. Figure 2:

 This figure is very similar to the one that appeared on the paper written by the authors’ team in 2019 (reference #22). The conceptualization figure must be a self-citation of their previous work, but the figure does not cite reference #22. It is recommended to specify the citation source even though the work is their own project.

5. Figure 2:

 I cannot understand the words “US child mortality” in the box at the third from the top at the right end. Are the authors going to study child mortality rate in the United States particularly?

Is the study design appropriate for the research question?

Yes

Is the rationale for, and objectives of, the study clearly described?

Yes

Are sufficient details of the methods provided to allow replication by others?

Yes

Are the datasets clearly presented in a useable and accessible format?

Yes

Reviewer Expertise:

Neonatology

I confirm that I have read this submission and believe that I have an appropriate level of expertise to confirm that it is of an acceptable scientific standard.

# Peer review: Reviewer response for version 1

**DOI:** 10.21956/gatesopenres.14951.r33242

**Published:** 2023-05-25

**Authors:** Jose R Duncan

**Affiliations:** 1 Department of Obstetrics and Gynecology, University of South Florida, Tampa, Florida, USA

I would like to congratulate the authors for creating this concise research protocol titled: “Study protocol for UNICEF and WHO estimates of global, regional, and national low birthweight prevalence for 2000 to 2020”. In this protocol, Krasevec and collaborators, designed a thorough protocol to report the low birthweight (LBW) prevalence for individual countries, regions, and globally.

  

- Is the rationale for, and objectives of, the study clearly described?

The authors described the importance and rationale for designing their study and the objective is clear and concise.

  

- Is the study design appropriate for the research question?

The study is thoroughly defined with clear search methodology, reporting methods, and use multiple ways to minimize and assess bias and confounding. Even though, the authors also acknowledge that it will be impossible to adjust for all biases.

  

- Are sufficient details of the methods provided to allow replication by others?

Yes.

  

- Are the datasets clearly presented in a useable and accessible format?

There are clear and complete data extraction methodology, but I could not see the datasets that will be use or have been already collected.

 The authors described thoroughly the methodology of how they will do this study. They try to minimize bias and confounding utilizing multiple interventions, these include:

- Well defined exclusion criteria.

- They utilize the WHO data quality review framework, to assess the quality of the data.

- They described a priori methodology to select covariates to be included in a model that includes multiple steps. They plan to corroborate covariates with UNICEF and WHO, they will also do a cluster analysis.

- They described in detail the how they will obtain the LBW prevalence and report their results.

- The authors plan to perform multiple sensitivity analyses to corroborate their LBW prevalence and use it to compare to previously used models to estimate previous LBW prevalence.

I will recommend the authors to consult an experienced statistician to verify that there are no other interventions that could improve their models.

 I will also caution of not including the countries with outlier LBW prevalence and will recommend the authors to present the complete data and calculate prevalence with and without those with LBW prevalence < 2% or greater than 40%.

 Lastly, I would like to see the authors include a statement acknowledging that a prospective design will have been ideal to assess the LBW, however, this methodology will take years to complete.

Is the study design appropriate for the research question?

Yes

Is the rationale for, and objectives of, the study clearly described?

Yes

Are sufficient details of the methods provided to allow replication by others?

Yes

Are the datasets clearly presented in a useable and accessible format?

Not applicable

Reviewer Expertise:

Obstetrics

I confirm that I have read this submission and believe that I have an appropriate level of expertise to confirm that it is of an acceptable scientific standard.
